# Early ART initiation among HIV-positive pregnant women in central Mozambique: a stepped wedge randomized controlled trial of an optimized Option B+ approach

**DOI:** 10.1186/s13012-015-0249-6

**Published:** 2015-04-30

**Authors:** James F Cowan, Mark Micek, Jessica F Greenberg Cowan, Manuel Napúa, Roxanne Hoek, Sarah Gimbel, Stephen Gloyd, Kenneth Sherr, James T Pfeiffer, Rachel R Chapman

**Affiliations:** Department of Global Health, University of Washington Schools of Medicine and Public Health, 1705 NE Pacific St.,, Seattle, WA 98195 USA; Health Alliance International (HAI), 1107 NE 45th St., Suite 350, Seattle, WA 98105 USA; Department of Family Medicine, University of Washington, Box 356390, Seattle, WA 98195 USA; Department of Anthropology, University of Washington, Box 353100, Seattle, WA 98195 USA; Beira Operations Research Center, Ministry of Health, Ponta Gea, Beira, Mozambique; Department of Family and Child Nursing, University of Washington, Box 355809, Seattle, WA 98195 USA

**Keywords:** Option B+, PMTCT, HIV, Stepped wedge randomized controlled trial, Implementation science, Mozambique, Value stream mapping, Care cascade analysis, Systems analysis, Quality improvement

## Abstract

**Background:**

Despite effective prevention strategies and increasing investments in global health, maternal to child transmission (MTCT) of HIV remains a significant problem globally, especially in sub-Saharan Africa. In 2012, there were 94,000 HIV-positive pregnant women in Mozambique. Approximately 15% of these women transmitted HIV to their newborn infants, resulting in nearly 14,000 new pediatric HIV infections that year. To address this issue, in 2013, the Mozambican Ministry of Health implemented the World Health Organization-recommended “Option B+” strategy in which all newly diagnosed HIV-positive pregnant women are counseled to initiate combination anti-retroviral therapy (ART) immediately upon diagnosis regardless of CD4 count and to continue treatment for life. Given the limited experience with Option B+ in sub-Saharan Africa, few rigorous pragmatic trials have studied this new treatment strategy.

**Methods:**

This study utilizes an initial formative research process involving patient and health care provider interviews and focus groups, workforce assessments, value stream mapping, and commodity utilization assessments to understand the strengths and weaknesses in the current Option B+ care cascade. The formative research is intended to guide identification and prioritization of key workflow modifications and the development of an enhanced adherence and retention package. These two components are bundled into a defined intervention implemented and evaluated across six health facilities utilizing a stepped wedge randomized controlled trial study design. The overall objective of this trial is to develop and test a pilot intervention in central Mozambique to implement the new Option B+ guidelines with high fidelity and increase the proportion of HIV-positive pregnant women in target antenatal clinics (ANC) who start ART prior to delivery and are retained in care.

**Discussion:**

This pragmatic study utilizes research strategies that have the potential to meaningfully improve the Option B+ care cascade in central Mozambique and to decrease the MTCT of HIV. This trial is designed to identify critical low-cost improvement strategies that can be bundled into a defined intervention. If this intervention has a measurable impact, it can be rapidly scaled up to other ANC in Mozambique and sub-Saharan Africa.

**Trial registration:**

ClinicalTrials.gov: NCT02371265.

## Background

HIV remains the leading cause of death globally among women of reproductive age [1-3]. In 2012, only 62% of pregnant women were tested for HIV and started on antiviral therapy [1]. An estimated 90% of the world’s 2.5 million children under 15 years old living with HIV are in sub-Saharan Africa [4]. Over 95% of pediatric HIV infections result from mother-to-child transmission (MTCT) [4]. The rate of MTCT of HIV is 20%–45%, 15%–30% transmission risk *in utero* or at delivery, and 5%–20% risk through breastfeeding overall, reaching 30%–60% in low- and middle-income countries (LMIC) [5,6]. Without treatment, half of all children living with HIV die by their second birthday [2]. Short courses of anti-retroviral (ARV) drugs started early in pregnancy or during labor can reduce the risk of *in utero* and peripartum HIV transmission two- to threefold [5]. In resource-poor settings where caesarean delivery is rarely available or safe, initial prevention of MTCT (PMTCT) efforts focused on reducing MTCT using ARV therapy during labor and delivery, promotion of exclusive breastfeeding for 6 months, and then complete breastfeeding cessation [7-10]. PMTCT includes testing and counseling, family planning counseling, provision of an appropriate anti-retroviral regimen for women and newborns, and support for safer infant feeding.

In Africa, a troubling pattern of loss to follow-up (LTFU) has emerged at each stage of the PMTCT “treatment cascade.” A 2004 study in South Africa showed a loss-to-follow-up rate of 85% at 12 months by HIV-exposed infants [11,12]. Data from Malawi in 2005 showed cumulative loss-to-follow-up rates of 55% as early as the 36th week of pregnancy, 68% at delivery, 70% at 1st postnatal visit, and 81% at the baby’s 6-month postnatal visit in rural district hospitals [13]. Data from Kenya reveal high drop-out rates as well: 31.5% of HIV-positive women do not return for their HIV test results, 53.6% of those who got their results did not enroll in an HIV clinic, and 80.7% do not return for delivery [12].

Mozambique’s 11.1% HIV prevalence rate and under-5 mortality rate of 87 per 1,000 live births are among the most severe in sub-Saharan Africa [1,14]. Mozambique began the scale-up of free national HIV care services in 2004 including combination anti-retroviral triple therapy (ART), and by 2012, an estimated 282,000 people had started ART [1,3]. The national PMTCT program, initiated in 2002 provides free HIV counseling and universal opt-out testing for pregnant women attending antenatal clinics (ANC) and maternities. Despite many successful efforts such as the scale-up of ART and the use of ARVs during labor and delivery for most HIV-positive pregnant woman, in 2012, Mozambique still reported over 77,000 AIDS-related deaths, 120,000 new HIV-infected individuals including 14,000 infants, and 1,600,000 adults and children living with HIV [1,3]. During 2012, there were an estimated 94,000 HIV-positive pregnant women in Mozambique and 79% received some form of ARV therapy (approximately 65,000 got zidovudine (AZT) and 10,000 ART) [1,3]. Despite this fact, 15% of HIV-positive pregnant women transmitted HIV to their newborn infants, resulting in the majority of the 14,000 pediatric HIV infections that year [1]. As a result, treating HIV-positive pregnant women with combination ART and not just AZT to prevent new pediatric HIV infections is a major strategic priority for the Mozambique Ministry of Health (MoH) [3].

In 2010, the World Health Organization (WHO) developed new treatment guidelines, termed “Option B,” that emphasized early initiation of ART in antenatal care for all HIV-positive pregnant women [15]. According to Option B, those with CD4 ≤ 350 cell/mm^3^ initiate ART as therapy for life, while those with CD4 > 350 start ART in ANC as prophylaxis and discontinue treatment after cessation of breastfeeding. In 2012, the WHO issued a programmatic update endorsing a third option termed “Option B+” in which HIV-positive pregnant women initiate ART during pregnancy regardless of CD4 count and continue treatment for life [16]. In sub-Saharan Africa, Option B+ was first piloted and then widely implemented in Malawi [17,18].

The new Option B+ approach has been adopted by the MoH in Mozambique and is in the adolescent phase of implementation. As in many African settings, numerous health system factors present major challenges to successful adoption of the guidelines [18,19]. In Mozambique, ANC and HIV testing coverage is high but there is substantial LTFU at successive stages in the treatment cascade, limited counseling for women, and many barriers to actively track those women lost to follow-up [20]. Early MoH data suggests significant challenges remain for retention in care and adherence for women started on ART via the new Option B+ framework in Manica and Sofala provinces and throughout Mozambique. The successful implementation of new Option B+ WHO guidelines therefore requires major streamlining of links among ANC, PMTCT, and ART services to successfully reduce the rates of pediatric HIV infection in Mozambique.

### Goals and objectives

The overall objective of this study is to develop and test a pilot intervention in central Mozambique to implement the new WHO guidelines with high fidelity and increase the proportion of HIV-positive pregnant women in target ANC clinics who start ART prior to delivery and improve retention in acre after 90 days. The intervention emphasizes a WHO-defined Option B+ approach; HIV-positive mothers will be referred for ART at the time they receive a positive HIV test result during their first ANC visit.

## Methods

### Trial design

The project utilizes an initial formative research process to understand inefficiencies in the current Option B+ care cascade, to guide identification and prioritization of key workflow modifications and the development of an enhanced adherence and retention package. These two components, described in detail below, are bundled into a defined intervention implemented and evaluated across six health facilities utilizing a stepped wedge randomized controlled trial (with the health facility as the unit of randomization).

### Study facilities and setting

**Figure 1:**
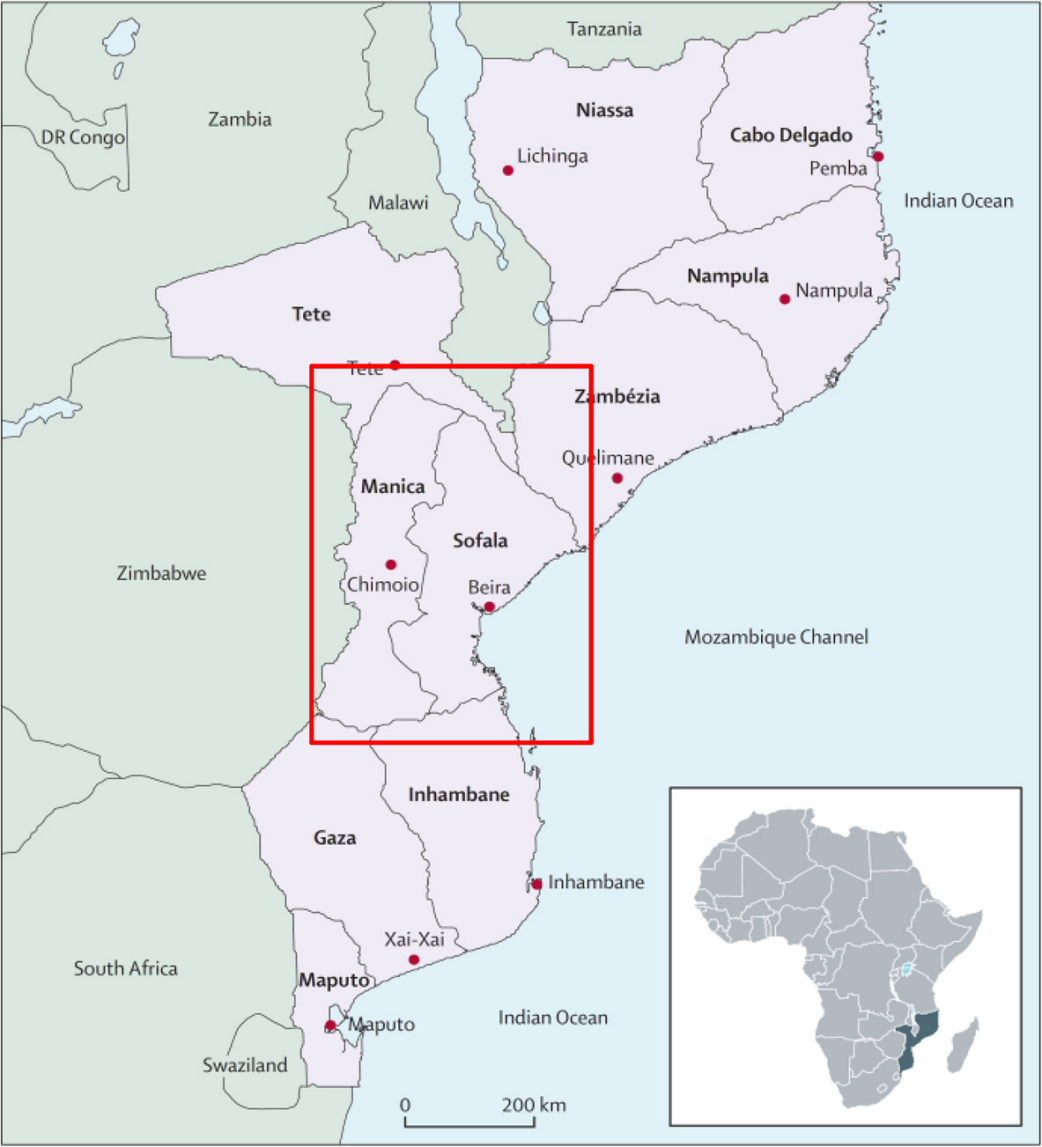
Map of Mozambique—provinces and provincial capitals [12].

As a facility-level intervention, the proposed “test-and-treat” Option B+ intervention is implemented through a stepped wedge design (described below) in six high-volume health centers providing PMTCT and ART services in the Mozambican national health system. These health centers serve communities along the highly populated Beira highway and railway transport corridor that passes through Sofala and Manica provinces (Figure 1), from the port city of Beira on the Indian Ocean to the Zimbabwe border [21].

Intervention sites include three health facilities in Sofala Province (Macarungo, Munhava, and Dondo) and three in Manica Province (Nhamaonha, 1° de Maio, and Gondola). All of these public health facilities provide the full range of PMTCT services, including HIV testing, access to CD4 testing (four out of six through transport to a central lab), and ART. ART has already been decentralized to ANC through MoH trainings. The intervention will be designed to enhance the new national MoH Option B+ ART policy in which all pregnant women attending their first ANC visit will be tested for HIV and, if positive, will be provided with first-line triple ART (TDF + 3TC + EFV, single daily fixed-dose combination) during that same visit, or within 14 days of the first visit and HIV test.

### Central Mozambique

In the central provinces of Sofala and Manica where the research is conducted, the provincial HIV prevalence rates of adults aged 15–49 years are among the highest in the country for women at 15.6% and 17.8%, respectively, and 14.8% and 12.6% for men [22]. The prevalence among pregnant women in 2009 was estimated at 18% [23,24]. HIV testing and treatment services in Manica and Sofala provinces have been scaled up with technical and financial support from Health Alliance International (HAI), a US non-profit affiliated with the University of Washington, School of Public Health. By early 2010, ART had been made available in 39 public sector sites [24]. In Manica, over 15,000 and, in Sofala, nearly 22,000 Mozambicans have started ART.

In 2010, PMTCT services were offered in routine antenatal care in 93 health facilities in Manica and 110 facilities in Sofala. First ANC visit coverage is consistently high at over 90% for Sofala and Manica provinces [24,25]. HIV testing at the first ANC visit has also attained consistently high rates of 90% in both provinces through the current opt-out testing program [25]. However, on average, women arrive late, between 19 and 25 weeks mean gestational age (unpublished data).

Prior to the introduction of Option B+, there were a number of documented challenges in the PMTCT care cascade: eligible HIV-positive pregnant women were referred to adult ART services but only about 20% started ART, only 29% of HIV-positive mothers received nevirapine during labor in central Mozambique, and only 60% of births occurred at a health care facility [22-24].

**Figure 2:**
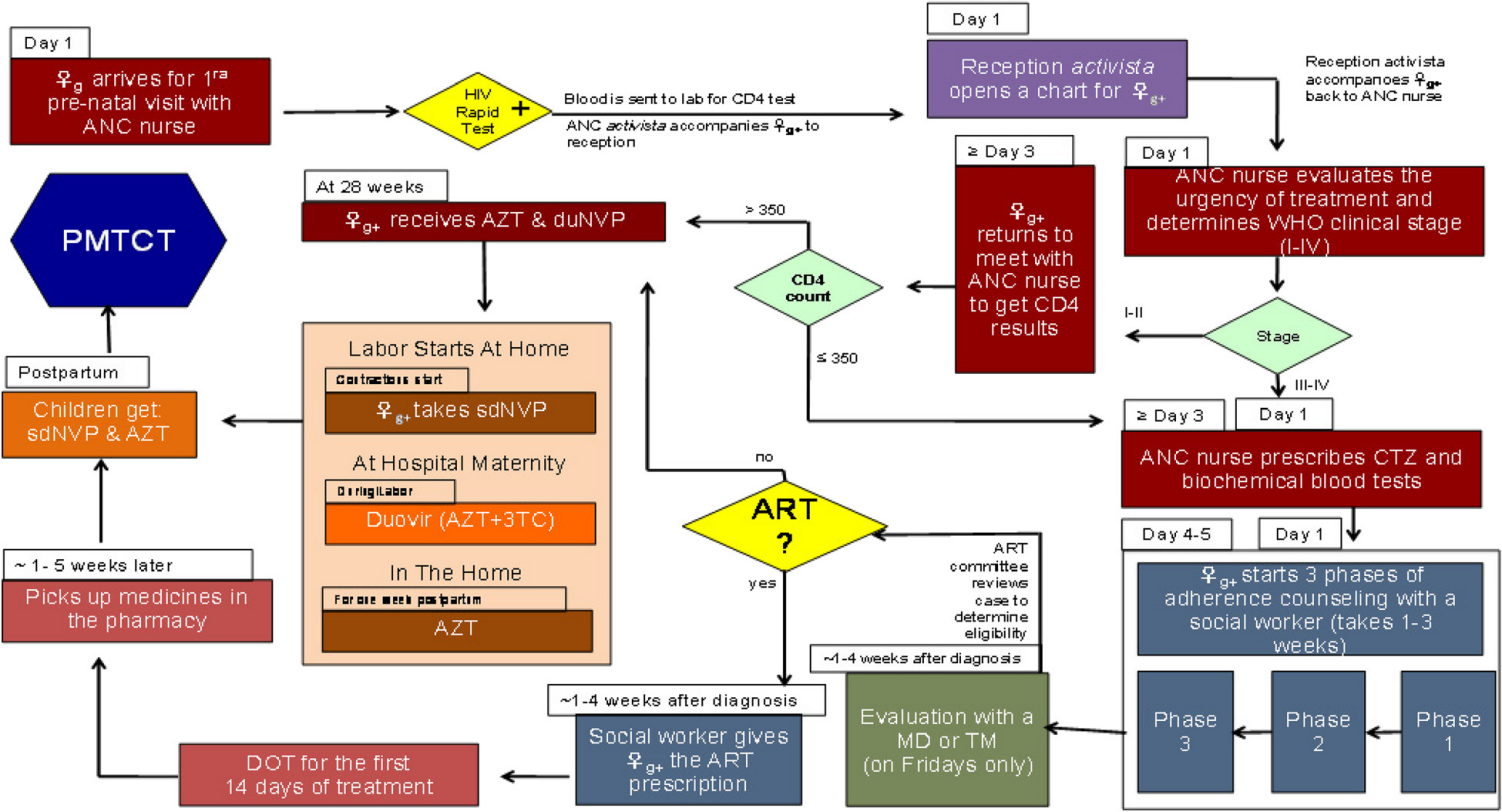
Munhava health center PMTCT patient flow.

Recent preliminary studies (2009–2010) conducted in the proposed research sites by UW and HAI researchers confirmed these previously reported barriers to care and sources of LTFU. A recent study of ART initiation and the PMTCT treatment cascade in seven large health centers in Manica and Sofala, which include two of the target sites included in this proposal (Munhava highlighted in Figure 2 and Nhamaonha), showed major LTFU and very small proportions of eligible positive mothers initiating ART [20].

**Figure 3:**
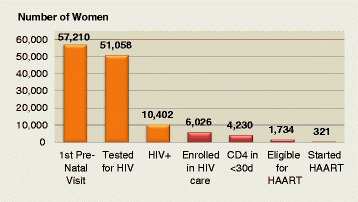
Aggregate flow cascade through seven health centers in Manica and Sofala provinces (2009).

This observational study, led by Chris Dodd and Jennifer Einberg, examined PMTCT LTFU under the previous protocol. The proportion that started ART varied significantly across the sites, but was low for each. Only about 40% of HIV-positive women actually received their CD4 results within 30 days of the first ANC visit, and only 40% of those eligible managed to start ART. Overall, only 19.3% of eligible women started ART across all the sites (see Figure 3). Province-wide program data from 2010 suggest similar patterns; only about 10% (8% in Manica and 12% in Sofala) of HIV-positive pregnant women started ART, while data suggests that an estimated 40% were actually eligible based on the protocol for ART initiation at that time [25].

Three recent qualitative studies by HAI researchers that focus on patient experience further corroborate sources of LTFU from the perspective of patients. Patients reported that requirement for multiple visits prior to ART initiation created major barriers for women because of transport costs and fears of unintentional HIV status disclosure causing stigmatization [26,27]. The last study revealed major gaps in understanding among HIV-positive women about steps to follow for care, benefits of care, and lack of system tools to support patient follow-up to ART and through postpartum visits. Women reported confusion about integrated ANC services, the purpose of the various tests and treatments provided (i.e., syphilis testing and treatment, IPT for malaria, and HIV and CD4 testing), and the need for follow-up visits [28].

Together, these studies indicate that PMTCT services and ART referrals require excessive repeat visits, provide insufficient information to patients, and lack patient tracking tools to ensure follow-up. The CD4 testing process and subsequent preparatory visits were associated with major delays and drop-offs in initiating ART and LTFU. Based on this evidence, interventions to increase earlier ART initiation and subsequent treatment adherence will require major streamlining of the process coupled with improved adherence counseling [29]. The Option B+ approach provides a model to achieve this streamlining if appropriately designed to capitalize on the strengths and recognize the constraints in the Mozambique heath system.

### Randomization

**Figure 4:**
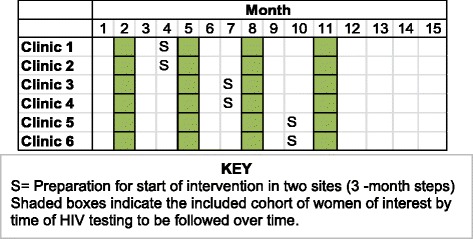
Stepped wedge design of the implementation of test-and-treat intervention in PMTCT programs in central Mozambique.

The introduction of the intervention in the study sites will occur in three steps following a stepped wedge design [30]. Prior to randomization, the six sites were stratified by province, and one site from each province (two total) was randomly selected to initiate the intervention at each of three stepped time points (months 5, 8, and 11; see Figure 4). Each step is separated by 3 months to allow an adequate number of people to be tested and initiate ART in each site (1.5 months, i.e., 1 month testing plus 14 days) and outcomes to be measured prior to the subsequent step (1.5 months, i.e., 45 days post-ART initiation). The implementation process includes staff training, integration of new adherence strategies, and intensive supervision and mentorship by the study staff and health facility managers to troubleshoot problems that arise.

#### Intervention description

- *Step one*: *formative research to improve the Option B*+ *protocol and model*
  During the first year of the project, researchers conducted formative research at the six selected sites to determine how to best adapt the Option B+ model to the six facilities. The research consists of these major activities:
  1. *Patient flow mapping*: Researchers mapped current patient flow patterns in the PMTCT treatment cascade and links to ART services at each of six target sites to identify differences and commonalities among the sites similar to the example from Munhava heath center presented in Figure 2. Future state flow maps have been created for each site to demonstrate new flow pathways required for Option B+ in collaboration with health center staff and managers. Health systems data for each site, including the monthly number of ANC visits, new ANC enrollees, new ANC enrollees tested for HIV, CD4 tests ordered and received, and the number of patients started on lifelong ART were collected to identify LTFU in the treatment cascade and ART initiation rates. These data were gathered from ANC registries, pharmacy registries, and ART clinic databases.
  2. *Workforce*: Patient volume in ANC and outpatient ART services was determined for each site during supervision visits. Staffing level, patient waiting times, and patient flow were examined for each site to identify workforce-related bottlenecks. Staffing levels and training needs required to manage new patient flows were identified for each site and incorporated into new patient flow mapping.
  3. *Flow mapping of materials and medications*: Flow and adequacy of key testing materials (HIV and CD4 testing materials), laboratory monitoring resources, and medications required for ART and other HIV care were examined for each health unit by assessing pharmacy and facility records and stock-out patterns. Current and future state flow mapping diagrams were produced for each site to indicate proposed medication, test materials, and patient flow changes and projections. Investigators also flow mapped laboratory monitoring protocols such as timing of biochemical panels, liver function testing, and complete blood count testing as determined by national MoH protocols. The pilot sites follow MoH protocol for initiating ART and monitoring patients following ART start.
  4. *Patient and health worker perspectives*: Separate qualitative focus group discussions (FGDs) were conducted with ANC patients, health workers, and counselors at ANC services, pharmacy, and outpatient services at each site to a) elicit perceptions of ART provision to pregnant women, b) solicit engagement in development of new Option B+ procedures, and c) identify key challenges to providing treatment. Researchers will also conduct individual interviews with facility directors and managers to support development of new pilot “Option B+” policies and protocols. The focus groups were conducted by a trained interviewer using open-ended, semi-structured interview guides, and we will plan to have ten patients and eight health care workers participate in FGDs or interviews at each site. Notes were taken by a trained notetaker and reviewed to identify themes, suggestions, and barriers to implementation.
  5. *Process measures*: Process measures appropriate to all sites were defined and data collection procedures established to measure fidelity to the core components of the intervention as it is implemented across the research sites. For example, measures may include proportion of patient records properly indicating timely counseling sessions or the first follow-up visit adherence rates in ANC.
- *Step two: develop the Option B+ intervention components: workflow modifications and an adherence and retention package*
  At the end of the formative research process in year 1, researchers developed key intervention materials together with the district and provincial directorates and the Beira Operations Research Center (CIOB), part of the Mozambican MoH, and review them for approval with health personnel at each site. Based on formative research findings produced in achieving the original specific aims 1 and 2, the following core components of the B+ study intervention have been designed and will be stepped in at the study sites to improve early retention in care and adherence:
  *Option B+ study core components*

I. *ANC workflow modification*
  A. Additional SMI nurse at each site to support clinical work but also focus on coordinating patient follow-up and use of SMS and phone calls.
  B. Workflow modification and specification at each site to define specific tasks for each nurse to optimize patient flow efficiency and reduce workload. Develop and distribute corresponding job aids.
  C. Additional community health workers (CHWs) at each site as needed who can help with patient file management, calling/texting patients, home visits, and patient tracking.

II. *Adherence and retention package*
  A. Facilitate monthly clinical chart review:
    Facility clinical director together with maternal child health (MCH) nurses conducts meetings.
  B. Adherence committee (AC):
  1. The committee consisting of MCH nurses and CHWs meets weekly to review individual patient adherence and coordinate follow-up strategies including active follow-up and texting.
  2. MCH nurses together with *activistas* coordinate intensified follow-up of each mother in the first weeks after the first initiation of ART in ANC, using texting and home visits to encourage return for the scheduled 7-day and subsequent visits during the first 30-day period.
  3. Following each AC meeting, the chief MCH nurse coordinates and ensures follow-up with patients by CHWs through systematic use of:
  SMS texting protocol developed to define messages and timing.
  Home visits with systematic supervision and follow-through using supervision checklist and AC review.
  C. Enhanced and intensified counseling:
    1. Modified messaging for the first counseling session with repeat counseling for each visit in first 30 days (7 days, 3 weeks, and before the first 30-day refill) and subsequent visits using content of the counseling over the next several months, based in part on data from formative research period and approved by MoH. Content includes enhanced counseling about partner notification and testing, side effects, and explanation of the ART/ANC process, with dissemination of intensified counseling messages to peer support groups.
  D. Supportive supervision: continuous quality improvement approach (CQI):
    1. Refresher ART and Option B+ training provided for all staff involved.
    2. The AC conducts monthly health facility ANC/ART data review to trouble shoot and resolve bottlenecks in patient flow.
    3. Option B+ checklist provided to clinical director at each HF to ensure quality in registry and patient file data collection, patient tracking processes registries properly filled out, and patient follow-up conducted.
    4. Option B+ checklist provided to MCH nurse for supervision of CHW patient follow-up.
  *Step three: stepped implementation of the Option B+ intervention bundle with concurrent impact evaluation*

Following the development of intervention components and before intervention implementation, in each facility as they are stepped in (designated as the “S” in Figure 4), a total of 25 staff (ANC nurses, receptionists, physicians, and physicians assistants or *técnicos*) participate in a 1-week training. The intervention bundle will be rolled out across the six intervention sites in three steps as described in Figure 4. The trainings focus on the MoH Option B+ protocol and the intervention core components as described above. Implementation trainings will include: a) refresher trainings on ART provision for MCH (SMI) nurses; b) new Option B+ policies, procedures, and drug regimes; c) laboratory testing protocols including CD4 count, hemograms, liver function testing, and biochemical panels as outlined by MOH PMTCT guidelines; d) new flow charts, job aids, treatment checklists, and data collection procedures; e) patient visit schedule for ANC and ART follow-up consultations through final postpartum visit; f) postpartum visit procedures for transfer and referrals of ART patients to outpatient services; and g) supervision procedures by physicians or physician assistants (*técnicos*) from outpatient services. Data will be collected as described below and will be evaluated in near real time to allow for concurrent impact evaluation.

### Implementation process measures

Throughout the intervention period, research teams will engage in process evaluation procedures to monitor the intervention and outcomes. These will include indicators for HIV testing, ART initiation, pharmacy refill rates, patient follow-up through visits and texts, adherence committee meetings, counseling sessions, and other key intervention activities. Researchers will conduct interviews with health workers to troubleshoot workflow challenges and bottlenecks and evaluate supervision practices. The process evaluation includes the following components:

- Weekly visits

- During the first 6 weeks of intervention at each facility: discussions with health workers (not requiring consent) to “fine tune” and adjust the intervention (while retaining core components).

- Monthly visits to health facilities

- Routine health facility data for outcome indicators collected monthly from registries and patient files.
- Discussions with health workers (not requiring consent) to “fine tune” and adjust the intervention (while retaining core components).
- Collect information concerning fidelity to core components as defined above.

- Quarterly visits

- Individual interviews (consented) with key health staff, including activists to assess intervention strengths and challenges.
- Focus group discussions with HIV+ mothers (consented) who have initiated ART to assess intervention strengths and challenges.

- Stepped wedge schedule

- Key outcomes measured and calculated per stepped wedge schedule collected from registries and patient files.

### Study timeline

Year 1 focused primarily on formative research. Year 2 included designing the intervention and seeking IRB and MoH approvals, and in year 3, the intervention will be rolled out following the stepped wedge design with concurrent evaluation of impact as outlined in Figure 4.

### Study measures and primary outcomes

We will measure early retention by calculating scheduled 30-day visit rates for pharmacy refill (up to 45 days from ART initiation) among women who initiate ART under the study intervention model and compare these rates to women starting ART before the intervention, while controlling for trends over time. We will also calculate 90-day adherence rates among women who initiate ART under the study intervention model and compare these rates to women starting ART before the intervention, while controlling for trends over time. We hypothesize that the proportion of women initiating ART during the intervention phase who return for their 30-day refill visit within 45 days will increase from 50% to at least 70%. We also hypothesize that the proportion of women initiating ART during the intervention phase who have 90-day ART adherence rates ≥90% based on pharmacy refill data will increase from 40% to a least 60%.

As a secondary, but critical, outcome, we will measure, monitor, and compare the proportion of HIV-positive pregnant women starting appropriate ART within 14 days of HIV testing before and after the intervention. Option B+ rollout data for ART initiation collected during the formative research period have varied widely among the six sites; however, MoH officials are confident that ART initiation rates will improve substantially and stabilize at a higher level using current protocols before the intervention begins, so follow-up remains the greater challenge. The intervention will seek to ensure that each site achieves at least 75% ART initiation (of mothers testing HIV-positive) within 14 days of HIV testing, and researchers will monitor and troubleshoot site performance throughout the research period. Study outcomes are summarized in Table 1.

**Table 1:** **Option B+ stepped wedge study outcomes measures**

**Study measure**	**Numerator/denominator**
1. First month ART pharmacy refill rates for newly diagnosed HIV-positive pregnant women	$\frac{\#\ \mathrm{of}\ \mathrm{newly}\ \mathrm{diagnosed}\ \mathrm{H}\mathrm{I}\mathrm{V}\hbox{-} \mathrm{positive}\ \mathrm{pregnant}\ \mathrm{women}}{\#\ \mathrm{of}\ \mathrm{newly}\ \mathrm{diagnosed}\ \mathrm{H}\mathrm{I}\mathrm{V}\hbox{-} \mathrm{positive}\ \mathrm{pregnant}\ \mathrm{women}\ \mathrm{that}\ \mathrm{refill}\ \mathrm{their}\ \mathrm{ART}\ \mathrm{prescription}\ \mathrm{within}\ 45\ \mathrm{days}\ \mathrm{of}\ \mathrm{diagnosis}}$
2. 90-day ART pharmacy refill rates for newly diagnosed HIV-positive pregnant women	$\frac{\#\ \mathrm{of}\ \mathrm{newly}\ \mathrm{diagnosed}\ \mathrm{H}\mathrm{I}\mathrm{V}\hbox{-} \mathrm{positive}\ \mathrm{pregnant}\ \mathrm{women}}{\#\ \mathrm{of}\ \mathrm{newly}\ \mathrm{diagnosed}\ \mathrm{H}\mathrm{I}\mathrm{V}\hbox{-} \mathrm{positive}\ \mathrm{pregnant}\ \mathrm{women}\ \mathrm{that}\ \mathrm{routinely}\ \mathrm{refill}\ \mathrm{the}\mathrm{ir}\ \mathrm{ART}\ \mathrm{medications}\ \mathrm{during}\ \mathrm{the}\ 90\ \mathrm{days}\ \mathrm{after}\ \mathrm{the}\mathrm{ir}\ \mathrm{diagnosis}}$
3. The percentage of newly diagnosed HIV-positive pregnant women that initiate ART within 14 days	$\frac{\#\ \mathrm{of}\ \mathrm{newly}\ \mathrm{diagnosed}\ \mathrm{H}\mathrm{I}\mathrm{V}\hbox{-} \mathrm{positive}\ \mathrm{pregnant}\ \mathrm{women}}{\#\ \mathrm{of}\ \mathrm{newly}\ \mathrm{diagnosed}\ \mathrm{H}\mathrm{I}\mathrm{V}\hbox{-} \mathrm{positive}\ \mathrm{pregnant}\ \mathrm{women}\ \mathrm{that}\ \mathrm{initiate}\ \mathrm{ART}\ \mathrm{within}\ 14\ \mathrm{days}\ \mathrm{of}\ \mathrm{their}\ \mathrm{diagnosis}}$

*ART* anti-retroviral therapy, *HIV* human immunodeficiency virus, *PMTCT* prevention of maternal to child transmission of HIV.

### Data sources

Research teams will measure patient ART adherence by extracting data from routine health records. There are three sets of patient health records system that researchers will have access to for each subject: 1) the ANC registry that is completed for every woman in her initial ANC consultation, 2) the HIV treatment registry and chart that is used for each patient who initiates ART, and 3) the refill records of the pharmacy. The data extracted from each of these sources will be consolidated into a single Microsoft Access database. Once consolidated in the new data set, the patients will be identified using a new study-specific identification number in the data set for analysis. Data extraction from registries is performed by trained study team members in each facility and compared for consistency. In cases of inconsistency, data collection will be repeated.

### Analysis

All data will be collected in Mozambique and analyzed using Stata SE (StataCorp, College Station, TX). We will compare outcomes in the pre- and post-intervention phases and will use an intention-to-treat analysis that adjusts for clustering by health facility and changes over time. Two analysis approaches will be considered.

First, if each measurement period has a similar number of observations per period per site, each site will contribute four outcome measurements for 30-day ART pharmacy refill rates over time (one for each step period described in Figure 4), and the outcome measures (proportions) will be collapsed into continuous numeric variables. We will then perform a linear regression analysis for repeated measure panel data, specifying our outcome as the dependent (continuous) variable and intervention status (yes/no) as the main independent variable, while adjusting for time and first-level autocorrelation between successive measures. Health facility will be specified as the panel to control for clustering by clinic.

If the number of observations does vary over time and between sites, we will do an alternative individual-level analysis that uses individual-level data with the outcome coded in a bivariate format (yes/no). We will then use logistic regression to determine the relationship between the outcome and the intervention period (pre vs. post), while controlling for time period and clustering by site.

The analysis will also focus on the calculation of 90-day ART adherence dichotomized into good/poor as described above, among all patients starting ART in the post-intervention phase. To determine if over 60% of those initiating ART have good adherence, we will calculate 95% confidence intervals using the binomial method and determine whether this interval includes (or is above) the goal of 60%. We will compare the proportion of patients with good 90-day adherence in the pre- vs. post-intervention phase, using the same stepped wedge methodology described above.

### Sample size

The power calculation for this study is based on the primary outcome (proportion of HIV-positive pregnant women starting ART who return within 45 days for the first 30-day refill) and based on published calculations for stepped wedge designs [30]. For this calculation, we estimate that at minimum 30 new HIV-positive women will initiate ART in each of the ANC centers for each 1-month measurement period and that the baseline proportion of women returning within 45 days is 50%, based on data from formative research. For a two-sided α of 0.05 and an estimated coefficient of variation (*k*) between facilities of 0.2, we would have 84.4% power to detect an expected increase in our post-intervention outcome of 20% (i.e., from 50% to 70%). The power changes minimally with varying coefficients of variation (i.e., 82.0% power with *k* = 0.4). To maintain at least 80% power, we would be still able to detect a difference of 19% from the baseline 50% (i.e., to 69%).

To determine the precision of our estimate for 90-day adherence post-intervention, we estimate that an average of 30 new HIV-positive women who initiate ART will be identified per ANC site for each 1-month measurement period. As there are 12 post-intervention periods under consideration, we estimate that 720 (30 × 12) will initiate ART. This denominator would allow a calculation of adherence that is within ±4% for adherence rates >50%.

### Ethics

The institutional review board of the Ministry of Health of Mozambique and the University of Washington approved this study. The trial is currently registered with ClinicalTrials.gov #NCT02371265.

### Trial status

Formative research at all six sites in central Mozambique began in March 2013. The intervention bundle was finalized in 2014. Implementation of the stepped wedge randomized controlled trial is in process.

## Discussion

This stepped wedge randomized controlled study is designed as a pragmatic trial to evaluate and strengthen the current Mozambican MoH support rollout of Option B+ in central Mozambique and to reduce high LTFU of HIV-positive pregnant women from PMTCT services and ART. This trial supports the development, implementation, and evaluation of a scalable bundled intervention designed to increase the number of pregnant women that are newly diagnosed with HIV at their first ANC visit that start ART therapy and that continue taking their HIV medications throughout their pregnancy. There have been limited evaluations of Option B+ efforts in sub-Saharan Africa—the introduction of Option B+ in Mozambique provides a unique opportunity for a rigorous randomized controlled stepped wedge trial to evaluate and strengthen this process.

This evaluation will help to highlight current strengths and shortcomings in the current PMTCT care cascade on a facility-level basis. If the bundled intervention is successful, it can be rapidly scaled up to other ANC sites in Mozambique and sub-Saharan Africa.

The start of Option B+ and the formative research component of this study have generated great interest both at participating facilities and within the Mozambican MoH. However, there have also been several challenges thus far including the evolving MoH PMTCT guidelines (this trial was originally designed to evaluate Option B vs Option B+), the high volume of patients ANC staff manage each day, the addition of new responsibilities for these staff, national elections, floods, and military conflict [31].

Despite these challenges, this innovative stepped wedge trial has the potential to significantly improve the care provided to HIV-positive pregnant women in central Mozambique and to reduce the likelihood that they will transmit HIV to their newborns. The results of the formative research and the intervention evaluation are likely to be of significant interest to local, national, and international PMTCT providers and health policy experts. If successful, this model of formative research, intervention development, and randomized stepped wedge intervention evaluation can be applied across other major health services (TB, malaria, non-communicable diseases) and may provide a practical example for how rigorous health systems research can occur in other low-income countries.

## Abbreviations

ANC: Antenatal care
ART: Combination anti-retroviral therapy
ARV: Anti-retroviral medication
AZT: Zidovudine
CD4: Cluster of differentiation 4 (laboratory test)
CHW: Community health worker
HIV: Human immunodeficiency virus
IRB: Institutional review board
LTFU: Lost to follow-up
MCH: Maternal child health
MoH: Ministry of Health
PCR: Polymerase chain reaction (laboratory test)
PMTCT: Prevention of mother-to-child HIV transmission
WHO: World Health Organization
